# American Academy of Dental Science

**Published:** 1893-08

**Authors:** 

**Affiliations:** American Academy of Dental Science


### AMERICAN ACADEMY OF DENTAL SCIENCE.

THE regular monthly meeting of the American Academy of Dental Science was held in the Boston Medical Library Association Rooms, March 1, 1893, at 7.30 P.M., President Brackett in the chair.

*President Brackett.*—The paper for the evening is by Dr. J. Adams Bishop. Subject, "Injuries and Diseases of the Mouth."[1] Owing to the absence of Dr. Bishop the paper will be read by his brother, Dr. H. F. Bishop.

DISCUSSION.

*President Brackett.*—Gentlemen, you have heard this most interesting and valuable paper. The subject is before you for discussion, and I will call on Dr. Clapp to make the opening remarks.

*Dr. Clapp.*—It is a matter of very great regret that our numbers are so small, because the historical facts in this paper are not only very interesting but very valuable.

I wish that a description of the manner of holding the parts in place while the impression is being taken had been more fully de-

---

[1] The paper by Dr. Bishop was read before the New York Odontological Society and also at the American Academy of Dental Science, and will be found in the May number, page 321.—ED.

scribed, for it seems to me that this must be the most difficult part of the whole operation of making these interdental splints.

The thoughts that the paper brings out show how much one class of specialists depends on another, and how one may assist the other, and how necessary the combined ingenuity and skill of all are to the best treatment of the injuries that happen to humanity. These injuries are of such importance to those who receive them that the very best skill is none too good for their treatment.

The amount of pain that may be saved by skilful as compared with that which must be endured by unskilful treatment is a matter of very great moment, and to us it is an occasion of pride that the great advance in the treatment of fractures of the jaw is due to the ingenuity of a dentist, and I hope that a complete and adequate record of this subject will be brought out at the Columbian Congress.

As regards the matter of ingenuity, particularly in original appliances, I am reminded of what was said at the annual meeting of the Harvard Odontological Society last Saturday night by Dr. Hunt. It was in this vein: that dentists have done a great deal in the matter of original research, and that the dental schools of this country are noted for the extent and value of what has been brought forward from an original stand-point. Whereas the medical schools follow largely the lead of the foreign schools, particularly those of the German and French. And he gave it as his opinion that the dental specialty of medicine, as he called it, was destined to be a leader rather than a follower in professional education, and that the field for young men with superior education and superior natural abilities was very great in dentistry. Just here let us think of the preparation that the dentist should have; we cannot say too often that the candidates for the dental profession should be thoroughly examined; that men of ordinary ability have no place at the present time in this profession; that the requirements are so exacting and so great that only those of superior natural ability can hope to take a place and to do the work that people with advanced ideas will require.

*Dr. H. F. Bishop.*—The suggestion that Dr. Clapp made is a good one, and I wish that my brother had described particularly the processes he uses for taking impressions. He is very expert in that work, and wherever he can he uses plaster, but there are cases where I am not sure that it can be used, and I am sorry that he has not described his methods. In the case of a fracture of the lower jaw, where there are teeth in both jaws, it is sometimes best to take an impression of one-half of the lower jaw at a time, and then accurately antagonize the two pieces with the model of the upper jaw.

*President Brackett.*—If Dr. Stevens will kindly take the chair for a few minutes I will say something on this subject.

*Dr. Brackett.*—It was my rare privilege to make Dr. Gunning's acquaintance in the year of my graduation from the Dental School, and to be an humble assistant of his in the practical treatment of a case of broken jaw. In the summer of 1873, Dr. Gunning was spending some weeks at Newport visiting some of his patients, and shortly after his arrival a girl of a dozen years of age was run over by a fashionable turn-out and sustained a fracture of the lower jaw. It began in the region of the right lateral incisor and extended downward at the expense of the posterior fragment.

A practical thing in connection with this case was that it had been seen by a physician of large experience, and the patient and her family had been told by him that no fracture was present.

This fact came to my mind as Dr. Bishop remarked that fractures are easy of diagnosis. It seems to me that they are not difficult if made by the dentist, but I question if the average general practitioner may always be depended on to make a ready and easy diagnosis in these cases. Dr. Gunning took the impression, but was without laboratory conveniences, and it was my privilege to be of some assistance to him and to Dr. Bishop, the preparer of this paper, who accompanied him, and who showed me in my office how to make the splint. After the return of these gentlemen to New York I was left in charge of the case. I have clearly in mind the procedures which were followed, and they would be practical in a large percentage of cases which we would be likely to meet. Dr. Gunning made no attempt whatever to reduce the fracture or put the two fragments of bone in relation to each other. That was before the days of modelling composition, and plaster was little used for impressions if there were teeth in the mouth. With ordinary beeswax in one of these cups was taken an impression, from which a plaster cast was made, which showed exactly the deranged condition of things. A study was made of the line which the fracture had taken, and with a saw the plaster cast of the lower arch was divided in about the direction that the fracture had separated it. A wax cast was also taken of the upper teeth.

All of the teeth were present in the mouth,—some temporary, some permanent, as you would expect to find in a patient of this age. With the upper arch as a guide, the lower teeth were brought to the right position with remarkable accuracy, just as we put together the models of a regulating case. Upon this correct model a vulcanite splint was made, proper antagonism being secured by imprinting the plaster model upon the wax.

Two-tailed ligatures were then passed about the necks of each lower temporary second molar, and the splint was applied, the fragments of the jaw coming readily into their right position. The ligatures, previously tied about the teeth, were brought out through holes drilled for them in the sides of the splint, and they were tied securely on top in a little groove so made that the ligatures might not be worn through in chewing. Almost immediately after the splint was put in place the pain was relieved, and, after a very short interval, the patient was doing a good amount of mastication.

The after-treatment consisted simply in keeping the parts as clean as possible with injections through holes made in the splint for that purpose.

Within a reasonable time a gratifying recovery took place.

From that time, for a period of fifteen or sixteen years, I think every broken jaw in and around Newport was in my hands for treatment. Among those which now come to my mind are a soldier in the detachment garrisoned at Fort Adams, who was run over by horses and a field-carriage; a captain of a whaling-vessel, who was thrown violently against the rail of his vessel during a storm; a young man who was struck by a boom on a yacht; another young man engaged in coupling cars, who received a very bad fracture.

Each of these cases had its own complication, requiring time, and in some instances the surgeon's aid, before they were overcome; but ultimately all except the soldier made good recoveries. The injury to his jaw was in the left angle and ramus, and permitted the rest of the jaw to fall backward and turn to the left. Extension relieved his suffering, and one of the problems was how to keep this extension continuous. It came to my mind to use a base-ball catcher's wire mask as a point of attachment for stout ligatures which had first been tied about the necks of strongly supported left lower molars. No interdental splint was used in his case, but I think an applicable form would have been the double one, enveloping the upper and lower teeth and screwed fast.

The poor man, for a long time previous to the accident, had been a victim to grave pulmonary and other disease; he had little vitality or recuperative power, and his death occurred some weeks after receiving the injuries.

The procedures followed in the first case that I described should be a sufficient guide to any dentist of ordinary ingenuity in managing an average case. That power of invention which is an essential endowment of every successful dentist will enable him to so modify his procedures as to be prepared for almost any case of the sort that may arise. Physicians and surgeons are ready to recognize the dentist's superior advantages and resources in the management of broken jaws, and to give him the full share of credit that he deserves.

*Dr. Cooke.*—Before we pass the subject I move that a vote of thanks be extended to Dr. J. Adams Bishop for his very valuable paper, and also to Dr. H. F. Bishop for his kindness in presenting the same, together with the interesting specimens, to the Academy.

A unanimous vote was passed.

*President Brackett.*—If the members have nothing more to say on this subject we will pass to "Presentation of Specimens and Incidents of Office Practice."

*Dr. Clapp.*—I want to speak of one incident of practice. Within a short time I have had two cases where the teeth have been cut away in order to fill the approximal surfaces, and now the teeth have come together at the necks, decay has reappeared around nearly all the fillings, and the amount of labor that is necessary on my part, and the patience and pain necessary on the part of these patients to get their mouths in a comfortable condition, is almost appalling. These cases show what seems to me a criminal practice of cutting away teeth to facilitate their filling.

In one of these mouths there is a peculiar condition. It was presented to me the day before yesterday. The four sixth-year molars were extracted many years ago, I should say before the eruption of the twelfth-year molars, as the second molars had taken the place of the first. Between the left superior second bicuspid and the adjacent molar is a swelling, and an abscess is threatened. I cannot find that any of the teeth on that side are dead, neither is there any apparent disease of the alveolus, and still there is a swelling like that caused by an abscessed tooth. I should like to know if any of you can tell me what causes it. It has occurred to me that possibly when this sixth-year molar was extracted, a sliver, the end of one of the roots, might have been left in, and that may be the cause of this lesion; that is the only explanation I can think of.

*President Brackett.*—I have had a parallel case that proved to be due to just what Dr. Clapp has suggested may be the cause in the case presented to him.

In the extraction at an early age of a right upper first molar a small fragment of a buccal root had been left. The contiguous teeth moved so as to be nearly in contact, and many years after the operation on the first molar the root fragment made a disturbance simulating an abscess of the second molar.

*Dr. Clapp.*—I have never had a case of this kind. I have had those of abscess arising from disease of the alveolus where there were no dead teeth, but in this the gum and alveolus is apparently healthy all about these teeth.

*Dr. Stevens.*—I had a case the other day that was very gratifying to me which I would like to tell you about. A boy, about fifteen years of age, who had never had any dental operation performed and who was very timid, came to the office, and was taken sick and had to go home after having almost nothing done. He came again, and I managed to cleanse his teeth, but it was a difficult task, as he was sick both in his head and stomach. He came again by appointment to have some filling done, and again became ill, and went home and had to lie in bed all that afternoon. It was simply a case of nerves, and I told the mother that one hour before he visited the office she should give him a five-grain phenacetin powder. She did so, and when he came I gave him another five-grain powder. The result was both surprising and gratifying, as the boy sat in the chair for over two hours without making any complaint or feeling any nervousness, and I succeeded in filling five cavities without any difficulty.

William H. Potter, D.M.D.,
*Editor American Academy of Dental Science.*

